# Oral Piercing in the Aspect of Periodontal and Dental Health—A Narrative Review

**DOI:** 10.3390/jcm15197582

**Published:** 2026-09-29

**Authors:** Dominika Cichońska, Jakub Hopke, Szymon Bieniek, Magda Balcerzak, Patryk Plaga, Paulina Adamska, Aleksandra Ciesielska, Aida Kusiak

**Affiliations:** 1 Department of Periodontology, Medical University of Gdańsk, M. Skłodowskiej-Curie 3c, 80-210 Gdańsk, Poland; 2 Student Research Group at the Department of Periodontology, Medical University of Gdańsk, M. Skłodowskiej-Curie 3c, 80-210 Gdańsk, Poland; 3 Division of Oral Surgery and Implantology, Medical University of Gdańsk, M. Skłodowskiej-Curie 3c, 80-210 Gdańsk, Poland

**Keywords:** oral piercing, lip piercing, tongue piercing, periodontal complications, dental trauma, gingival recession

## Abstract

Oral piercing is an increasingly popular form of body modification, particularly among adolescents and young adults. Despite its aesthetic and cultural appeal, growing evidence indicates that oral piercings have been associated with a range of oral and periodontal complications. This literature review examines the effects of oral piercing on periodontal tissues and summarizes the associated risk factors and preventive measures. The available evidence demonstrates that tongue and lower lip piercings are most associated with periodontal damage due to chronic mechanical trauma and bacterial plaque accumulation around oral jewelry. The principal periodontal complications include gingival recession, gingival inflammation, clinical attachment loss, periodontal pocket formation, and localized alveolar bone loss. In addition, oral piercings have been linked to tooth fractures, enamel wear, mucosal lesions, infections, hypersensitivity reactions, and, in rare cases, systemic complications such as infective endocarditis. The severity of these complications is influenced by factors including the duration of jewelry wear, jewelry design and material, oral hygiene, smoking, and individual periodontal characteristics. Current evidence suggests that many of these adverse outcomes can be minimized through appropriate patient education, the use of biocompatible and well-fitted jewelry, meticulous oral hygiene, and regular dental examinations. Although the existing literature consistently reports an association between oral piercing and periodontal complications, further longitudinal studies are needed to better define long-term outcomes and strengthen evidence-based clinical recommendations.

## 1. Introduction

Body piercing is one of the oldest forms of body modification and has been practiced for thousands of years in numerous civilizations for cultural, religious, spiritual, and social purposes. Historically, oral and facial piercings were used to symbolize tribal affiliation, social status, rites of passage, bravery, and religious devotion. In modern society, however, oral piercing has evolved primarily into a form of self-expression, aesthetic enhancement, and personal identity, particularly among adolescents and young adults [1,2,3]. Advances in piercing techniques, the growing popularity of body art, celebrity influence, and widespread exposure through social media have contributed to the increasing acceptance and prevalence of oral piercings worldwide over the past two decades [3,4].

Oral piercing involves the placement of jewelry through the soft tissues of the oral cavity or perioral region. The tongue, lower lip (labret), upper lip, labial frenulum, cheeks, and uvula are among the most pierced sites, with tongue and lower lip piercings accounting for most of procedures [5,6]. Jewelry used for these piercings typically includes barbells, captive bead rings, studs, and labret posts made from materials such as titanium, surgical stainless steel, niobium, acrylic, or gold. Although these materials are selected for their durability and biocompatibility, their continuous contact with oral tissues and teeth may predispose wearers to various local and systemic complications [7].

The prevalence of oral piercing varies considerably among countries and populations owing to differences in cultural practices, age distribution, and study methodology. Epidemiological studies have reported prevalence rates ranging from less than 1% in the general population to approximately 10% among adolescents and young adults, with the highest prevalence consistently observed in individuals between 18 and 30 years of age [2,8]. Women generally demonstrate a slightly higher prevalence of oral piercing than men, although this difference varies across geographical regions and social groups [6].

The motivations for obtaining an oral piercing are multifactorial and involve psychological, social, cultural, and aesthetic influences. Common reasons include self-expression, enhancement of physical appearance, individuality, fashion, peer influence, curiosity, and identification with particular social or cultural groups [2,6]. Some individuals perceive oral piercings as symbols of independence or rebellion against traditional social norms, whereas others report increased self-confidence and improved body image following the procedure [1]. Tongue piercings have also been associated with perceived enhancement of sexual experience, although scientific evidence supporting this belief remains limited [5]. Despite these motivations, numerous surveys indicate that many individuals are inadequately informed about the potential oral and systemic risks before undergoing the procedure [4,6].

Although oral piercing is generally regarded as a cosmetic procedure, increasing scientific evidence demonstrates that it may have significant implications for oral and systemic health. The oral cavity presents a unique environment characterized by constant mechanical function, abundant microbial flora, and continuous exposure to saliva, all of which influence the healing process and increase the potential for complications [5]. Oral jewelry acts as a foreign body that may repeatedly traumatize the surrounding soft and hard tissues while simultaneously providing additional surfaces for bacterial biofilm accumulation. Consequently, oral piercings have been associated with numerous complications, including gingival recession, gingival inflammation, clinical attachment loss, periodontal pocket formation, localized alveolar bone loss, tooth fractures, enamel wear, mucosal ulceration, infections, hypersensitivity reactions, speech disturbances, and, in rare cases, life-threatening systemic infections such as infective endocarditis [6,8,9].

This review narrows its focus to periodontal tissues because periodontal complications represent one of the most clinically significant concerns. Continuous mechanical trauma generated by tongue and lip jewelry, combined with persistent plaque accumulation and chronic inflammation, can initiate a cascade of periodontal changes that progress from gingival inflammation to connective tissue attachment loss and alveolar bone resorption [5]. The severity of these changes is influenced by numerous factors, including the location of the piercing, duration of jewelry wear, jewelry design and material, oral hygiene practices, smoking status, periodontal phenotype, and individual host susceptibility [7,9].

Given the increasing popularity of oral piercing and the growing body of evidence linking this practice to periodontal disease, it is essential for dental professionals to understand its biological effects and clinical implications. This literature review aims to summarize the current evidence regarding the impact of oral piercing on periodontal tissues, discuss the underlying mechanisms responsible for periodontal destruction, identify the major risk factors associated with adverse outcomes, review other oral and systemic complications, and highlight evidence-based preventive strategies that can reduce morbidity and preserve long-term oral health. Although previous systematic reviews have established an association between oral piercings and adverse periodontal outcomes, the underlying evidence remains heterogeneous and is distributed across observational studies, case series, and case reports. Moreover, existing syntheses have largely focused on the occurrence and magnitude of periodontal complications or on oral complications more broadly, with less emphasis on integrating patterns of periodontal injury with their proposed mechanisms, relevant risk modifiers, and practical preventive considerations. An updated, clinically oriented synthesis integrating these dimensions may therefore help translate the available evidence into more comprehensive risk assessment, patient counselling, prevention, and clinical management.

## 2. Literature Search Strategy

A comprehensive literature search was conducted to identify studies evaluating the impact of oral piercing on periodontal tissues and oral health. Electronic databases, including PubMed, Scopus, Web of Science and Google Scholar, were searched for articles published between 2000 and 2025. The search strategy combined Medical Subject Headings (MeSH) terms and free-text keywords using Boolean operators (AND, OR). The primary search terms included oral piercing, tongue piercing, lip piercing, body piercing, periodontal tissues, gingival recession, clinical attachment loss, periodontal pocket, gingival inflammation, alveolar bone loss, oral complications, oral health, and prevention. Additional relevant studies were identified through manual screening of the reference lists of eligible articles. Only publications written in English were considered. Systematic reviews, meta-analyses, randomized and non-randomized clinical trials, cohort studies, case–control studies, cross-sectional studies, and clinically relevant case reports that investigated the relationship between oral piercings and periodontal health were included in this review. Case-based evidence was considered particularly when addressing uncommon or severe complications for which comparative studies were unavailable. Animal and in vitro studies, conference abstracts lacking sufficient full-text information, editorials and commentaries without relevant clinical data, non-English publications, duplicate publications, and studies not addressing oral/perioral piercing or outcomes relevant to the scope of this review were excluded. The selected studies were synthesized to provide a comprehensive overview of the current evidence regarding the periodontal, dental, and general effects, associated risk factors, oral complications, and preventive measures related to oral piercing.

## 3. Effects of Oral Piercing on Periodontal Tissues

Oral piercings have been consistently associated with a variety of adverse effects on periodontal tissues, primarily resulting from chronic mechanical trauma and increased bacterial plaque accumulation around oral jewelry. The continuous contact between tongue or lip jewelry and the gingival tissues may initiate inflammatory responses that, over time, may contribute to irreversible periodontal damage. The most frequently reported periodontal complications include gingival recession, gingival inflammation, clinical attachment loss, periodontal pocket formation, and localized alveolar bone loss [6,8]. The severity of these changes depends on several factors, including the location and duration of the piercing, jewelry design and material, oral hygiene practices, and individual periodontal susceptibility [9]. Understanding these effects is essential for early diagnosis, prevention, and appropriate management of periodontal complications in individuals with oral piercings.

Despite the relatively consistent association reported between oral piercings and adverse periodontal findings, the strength of the available evidence should be interpreted with caution. Much of the primary literature consists of small cross-sectional or case–control studies, case series, and, for less common outcomes, individual case reports. These designs are useful for identifying clinically relevant associations but generally cannot establish temporality or causality. Furthermore, heterogeneity in piercing location, jewelry type and material, duration of wear, oral hygiene practices, smoking status, and periodontal assessment methods complicates direct comparison across studies and may introduce residual confounding. Consequently, although the available evidence supports an association between oral piercing and gingival recession, attachment loss, periodontal pocketing, gingival inflammation, and alveolar bone changes, the magnitude and causal nature of these relationships remain incompletely defined. Larger prospective longitudinal studies using standardized periodontal outcomes and appropriate control groups are needed to clarify these associations and identify the factors that modify periodontal risk.

### 3.1. Effect of Oral Piercing on Gingival Recession

Gingival recession is widely recognized as the most prevalent periodontal complication associated with oral piercings [8,9,10]. It is characterized by the apical displacement of the gingival margin beyond the cementoenamel junction, resulting in exposure of the root surface. This condition not only compromises esthetics, particularly in the anterior region, but also predisposes affected teeth to dentin hypersensitivity, root caries, cervical abrasion, plaque retention, and, in severe cases, progressive periodontal attachment loss. Unlike gingivitis, gingival recession is largely irreversible without periodontal plastic surgery, making prevention and early recognition particularly important [11].

The primary mechanism responsible for gingival recession in pierced individuals might be chronic mechanical trauma produced by repeated contact between the jewelry and the gingival tissues. During normal oral functions such as speaking, chewing, swallowing, and parafunctional habits, metal barbells or studs repeatedly strike or rub against the gingival margin, producing continuous microtrauma that eventually leads to soft tissue breakdown [5]. The severity of tissue damage might depend on the frequency and magnitude of this trauma, with patients who habitually play with their jewelry by moving or biting it demonstrating a significantly greater risk of gingival injury. The smooth but rigid metallic surface of oral jewelry can repeatedly compress and abrade the gingival tissues, resulting in localized inflammation followed by gradual apical migration of the gingival margin [8,9].

The location of the piercing strongly influences the pattern and severity of gingival recession, that are observed in adjacent periodontal tissues [12].

In addition to direct mechanical injury, oral piercings may indirectly contribute to gingival recession by promoting plaque accumulation and chronic inflammation. The jewelry provides additional retentive surfaces for bacterial biofilm formation and may interfere with effective tooth brushing and interdental cleaning. Increased plaque accumulation may lead to persistent gingivitis, which weakens the gingival tissues and increases their susceptibility to mechanical trauma [6]. Furthermore, recurrent mucosal ulceration surrounding the piercing may impair normal tissue healing, thereby accelerating periodontal soft tissue destruction in susceptible individuals [6].

### 3.2. Effect of Oral Piercing on Clinical Attachment Loss

Clinical attachment loss (CAL) is an important indicator of periodontal destruction and represents the cumulative loss of connective tissue attachment and supporting periodontal structures around a tooth. Unlike gingival inflammation, which is often reversible with appropriate plaque control, clinical attachment loss reflects permanent periodontal damage and is considered a key diagnostic parameter in periodontitis [13]. Oral piercings, particularly tongue and lower lip piercings, may contribute to localized clinical attachment loss through a combination of chronic mechanical trauma, persistent inflammation, and plaque accumulation [8]. Although clinical attachment loss is less frequently reported than gingival recession, it is of greater clinical significance because it indicates irreversible destruction of the periodontal attachment apparatus and may ultimately compromise tooth stability if left untreated [8].

The principal mechanism underlying attachment loss associated with oral piercings could be a repetitive mechanical injury that initiates an inflammatory response characterized by infiltration of inflammatory cells and the release of cytokines and matrix metalloproteinases (MMPs), which promote degradation of collagen fibers and connective tissue. Over time, persistent trauma may result in apical migration of the junctional epithelium and progressive loss of clinical attachment. This process is particularly evident when the traumatic stimulus remains present for prolonged periods and is exacerbated by inadequate oral hygiene [14].

The accumulation of dental plaque around tongue or lip jewelry increases the risk of chronic gingival inflammation, which, when combined with mechanical trauma, accelerates periodontal tissue destruction [6]. Persistent inflammation stimulates the production of inflammatory mediators such as interleukin-1β, tumor necrosis factor-α, and prostaglandin E_2_, which contribute to connective tissue degradation and promote osteoclast-mediated resorption of the supporting alveolar bone [15,16,17,18]. Consequently, attachment loss associated with oral piercings is considered multifactorial, with mechanical trauma from piercing jewelry and local plaque-associated periodontal inflammation potentially contributing to periodontal tissue destruction [5,8,9,12,19,20].

The severity and distribution of clinical attachment loss are influenced by the location of the piercing. Lower lip piercings predominantly affect the labial surfaces of the mandibular incisors and canines, where repeated contact between the jewelry and the gingival margin is associated with localized periodontal destruction [11,12,21,22,23]. Tongue piercings, in contrast, are more commonly associated with attachment loss and gingival recession affecting the lingual surfaces of the mandibular anterior teeth, consistent with repeated contact of the tongue barbell with these tissues during oral function [5,19,20,23,24]. Clinical studies have reported increased probing depths, clinical attachment loss, gingival recession, and other adverse periodontal parameters at sites associated with oral piercings compared with control or unaffected sites [20,23,25,26]. These findings support a predominantly localized effect associated with the position and mechanical interaction of the jewelry with adjacent periodontal tissues [9].

### 3.3. Effect of Oral Piercing on Periodontal Pocket Formation

Periodontal pocket formation is a significant periodontal complication that may develop in individuals with oral piercings as a consequence of chronic mechanical trauma, bacterial biofilm accumulation, and sustained inflammatory responses [9,26,27,28]. A periodontal pocket is characterized by pathological deepening of the gingival sulcus associated with loss of periodontal attachment and apical migration of the junctional epithelium [18,29]. Although periodontal pocket formation is reported less frequently than gingival recession, evidence indicates that oral piercings—particularly tongue and lower-lip piercings—may be associated with localized increases in probing pocket depth (PPD), especially at teeth adjacent to the jewelry [8,9,20,21,23,25,26]. The development of periodontal pockets is clinically important because deeper pockets provide an ecological environment favorable to dysbiotic subgingival biofilms, while simultaneously making effective plaque removal more difficult and potentially promoting progressive periodontal destruction [18,30].

The pathogenesis of periodontal pocket formation associated with oral piercings seems to be likely multifactorial. Repeated mechanical contact from barbells, studs, or other jewelry may traumatize adjacent gingival and periodontal tissues [5,19,27,28]. When mechanical injury occurs in the presence of persistent microbial challenge, the local inflammatory response involves recruitment of neutrophils, macrophages, lymphocytes, and other immune cells [18,31]. Pro-inflammatory mediators including interleukin-1β (IL-1β), tumor necrosis factor-α (TNF-α), and prostaglandin E_2_ (PGE_2_) contribute to periodontal tissue destruction and regulate pathways involving matrix metalloproteinases, which participate in degradation of the extracellular matrix and periodontal collagen [18,30,32,33]. Progressive loss of connective-tissue attachment permits apical extension of the junctional/pocket epithelium and contributes to increased probing depth and periodontal pocket formation [29,30].

Barbells, rings, and studs may create additional plaque-retentive areas allowing microorganisms to colonize both the jewelry and surrounding oral tissues, particularly when oral hygiene is inadequate [6,24,34]. Microbiological investigations have demonstrated that tongue-piercing sites and jewelry can harbor potentially periodontopathogenic microorganisms, suggesting that oral piercings may act as local microbial reservoirs [24,35,36]. With increasing biofilm maturation and periodontal inflammation, the microbial community may undergo ecological dysbiosis characterized by an increased abundance and activity of anaerobic, proteolytic, and inflammation-associated microorganisms [37,38].

### 3.4. Effect of Oral Piercing on Gingival Inflammation

Gingival inflammation is among the periodontal soft-tissue complications observed in association with oral piercings and may present clinically with erythema, edema, increased gingival crevicular fluid, bleeding on probing (BOP), and tenderness as a consequence of microbial biofilm challenge and local tissue irritation [5,8,9,20,26]. Plaque-induced gingivitis is generally reversible following effective disruption of the dental biofilm and control of local contributing factors; however, persistent inflammation in susceptible individuals may contribute to progression toward periodontal attachment loss when accompanied by the biological conditions necessary for periodontitis [39,40]. In oral-piercing users, clinical investigations have reported alterations in periodontal parameters including plaque accumulation, gingival inflammation, BOP, probing depth, and clinical attachment level, although findings vary among studies and piercing types [9,12,20,24,26].

The development of gingival inflammation in individuals with oral piercings is likely multifactorial, involving microbial biofilm accumulation together with mechanical irritation from the jewelry. Inadequate cleaning of the jewelry and adjacent tooth surfaces may therefore increase the local microbial burden [6]. As plaque-associated gingival inflammation develops, microbial and host-derived signals induce vasodilation, increased vascular permeability, leukocyte recruitment, and production of inflammatory mediators including IL-1β, IL-6, TNF-α, and PGE_2_. These inflammatory pathways contribute to the characteristic clinical manifestations of gingivitis, including erythema, edema, and bleeding on probing [18].

In addition to microbial plaque, repeated mechanical contact between oral jewelry and adjacent gingival tissues may contribute to localized irritation. Thus, plaque-associated inflammation and repeated mechanical irritation may coexist at piercing-adjacent sites and provide complementary mechanisms for maintaining localized periodontal inflammation [5,19,20,22,23].

The distribution of periodontal inflammatory changes may also be influenced by piercing location. Clinical studies using site-specific, case–control, or split-mouth comparisons support the predominantly localized nature of many piercing-associated periodontal alterations [12,22,26].

### 3.5. Effect of Oral Piercing on Alveolar Bone Loss

Alveolar bone loss is among the most serious periodontal complications reported in association with oral piercings because it represents destruction of the osseous component of the tooth-supporting apparatus. Although gingival recession is reported more frequently, localized alveolar bone loss has been documented clinically and radiographically in patients with tongue and lower-lip piercings [8,9,26,27,41,42]. Severe cases may be accompanied by increased probing depth, clinical attachment loss, and reduced periodontal support and may require surgical or regenerative periodontal treatment [43].

The mechanisms underlying piercing-associated alveolar bone loss are also likely multifactorial [5,19,27,42]. Where plaque-associated periodontal inflammation is also present, microbial challenge can stimulate innate and adaptive immune responses involving neutrophils, macrophages, lymphocytes, and other cells, with increased production of inflammatory mediators such as IL-1β, IL-6 (interleukin-6), TNF-α and PGE_2_ [18,32]. These mediators can promote an osteoclastogenic environment, in part through alteration of the RANKL (Receptor Activator of Nuclear Factor-κB Ligand)/osteoprotegerin balance and increased differentiation and activation of osteoclasts, thereby contributing to alveolar bone resorption [16,32,44,45]. Thus, severe osseous destruction at piercing-associated sites may reflect the combined influence of localized mechanical injury and inflammatory periodontal processes rather than mechanical trauma alone. Persistent microbial challenge adjacent to susceptible periodontal tissues may sustain local inflammation and, where periodontitis develops, contribute to destruction of the periodontal ligament and alveolar bone.

The distribution of severe periodontal destruction associated with oral piercing is often localized to tooth surfaces in close anatomical relationship to the jewelry. Radiographic case reports have documented localized alveolar bone loss adjacent to oral jewelry, supporting an important role for site-specific mechanical exposure [26,27,41,42].

**Figure 1:**
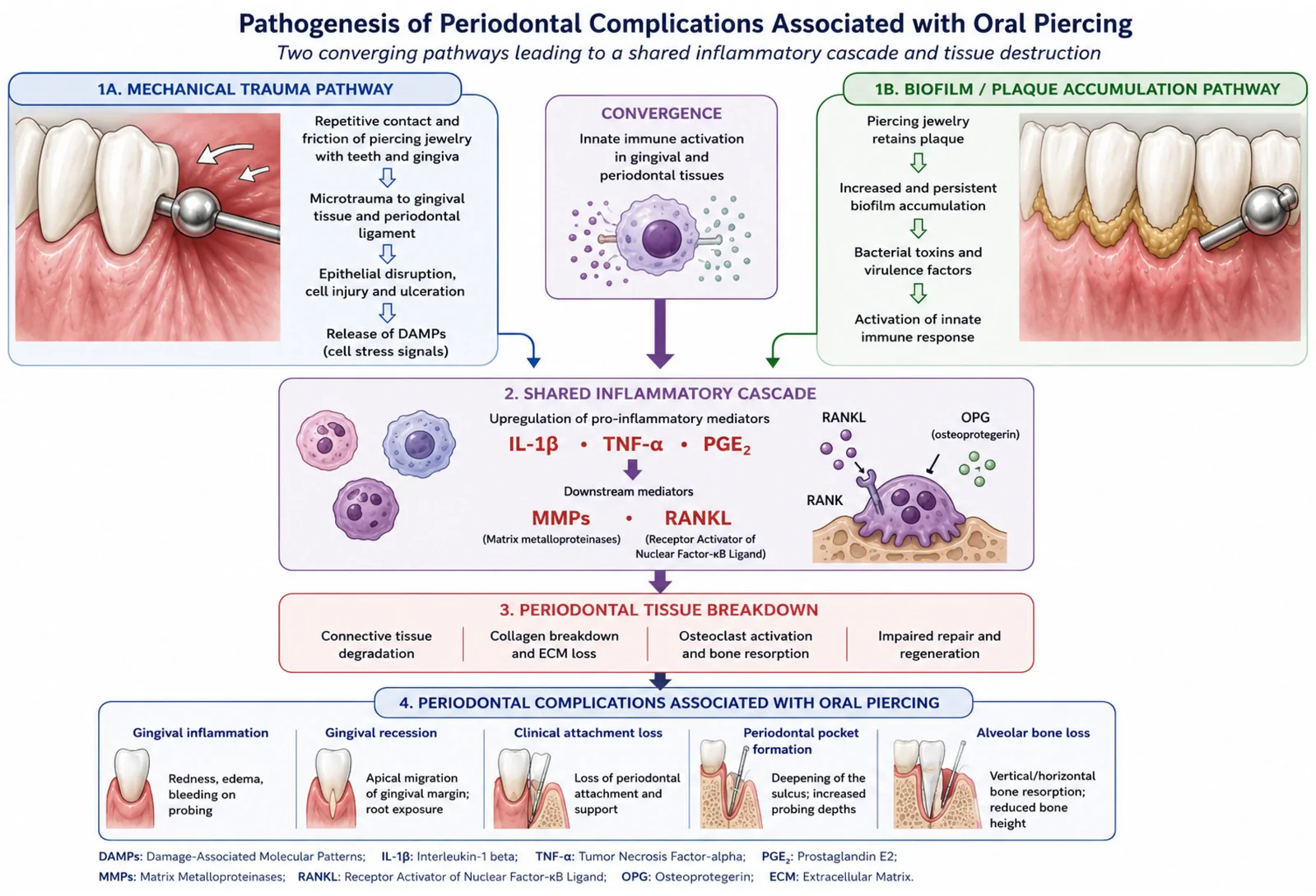
A schematic pathogenesis of periodontal complications related to oral piercing.

The summarized impact of oral piercings on periodontal tissues is presented in Table 1. A schematic pathogenesis of periodontal complications related to oral piercing is presented on Figure 1.

## 4. Factors That Are Associated with Periodontal Damage Related to Oral Piercing

The severity of periodontal damage associated with oral piercings might be influenced not only by the presence of the piercing itself but also by a combination of jewelry-related, patient-related, behavioral, and biological factors. While many individuals with oral piercings may experience only minor soft tissue irritation, others develop significant periodontal complications, including gingival recession, clinical attachment loss, periodontal pocket formation, and localized alveolar bone loss. The variability in clinical outcomes reflects the multifactorial nature of periodontal destruction, in which chronic mechanical trauma interacts with host susceptibility, oral hygiene practices, and environmental risk factors [8,9]. Understanding these risk factors is essential for identifying individuals at increased risk and implementing preventive strategies before irreversible periodontal damage occurs.

One of the factors associated with the development and severity of periodontal complications in individuals with oral piercings is the duration of jewelry wear. Several clinical studies have reported that prolonged exposure to tongue or lip jewelry is associated with a greater prevalence and/or severity of localized periodontal alterations, particularly gingival recession and attachment loss [19,20,23,35,46]. Campbell et al. [19] demonstrated an association between duration of tongue-piercing wear, barbell characteristics, and lingual gingival recession, suggesting that cumulative exposure to the jewelry may increase the likelihood of periodontal injury. Similarly, studies of lip piercings have identified duration of wear and the position of the jewelry relative to the gingival tissues as potential contributing factors to localized periodontal defects [23,35]. Repeated contact between oral jewelry and adjacent periodontal tissues over time may produce cumulative mechanical irritation, particularly when unfavorable jewelry dimensions, habitual manipulation, plaque accumulation, or susceptible periodontal anatomy are also present [5,6,8]. Consequently, longer piercing duration appears to increase cumulative exposure to local mechanical and microbial challenges and may contribute to progressively greater periodontal deterioration in susceptible individuals, although the magnitude of this association varies among studies [8,9]. Furthermore, multiple oral piercings or the simultaneous presence of tongue and lip jewelry increase the total area exposed to trauma and may therefore increase the overall risk of periodontal injury. However, less common piercing sites and configurations are represented predominantly by small observational studies and case reports. Consequently, the evidence is currently insufficient to establish reliable periodontal risk profiles for each specific piercing type. This imbalance in the literature should also be considered when comparing sites: more frequently studied piercing configurations may appear to carry greater risk simply because their complications have been more extensively documented [20,23,35].

The design, size, and material of the jewelry also play a crucial role in determining the extent of periodontal damage. Long barbell stems, oversized jewelry, and heavy metallic ornaments permit greater movement within the oral cavity, increasing the frequency and intensity of contact with the gingival tissues during speaking, chewing, and swallowing [19]. This repeated impact amplifies mechanical trauma and accelerates soft tissue destruction. Piercing type and jewelry configuration should also be distinguished from anatomical location. Barbells are commonly used for tongue piercings, whereas labret studs or similar devices are frequently used for lip piercings. Longer stems or oversized components may theoretically increase mobility and tissue contact, whereas poorly fitted intraoral components may maintain direct pressure against the gingival margin. Some observational studies have reported relationships between jewelry characteristics and periodontal or dental findings; however, these variables are closely correlated with piercing location and duration and have not been consistently evaluated in adequately adjusted multivariable analyses. Consequently, jewelry design should presently be regarded as a potential modifying factor rather than an independently established causal determinant. However, appropriately fitted jewelry with shorter stems reduces unnecessary movement and minimizes tissue injury. Jewelry manufactured from rough or poorly polished materials further increases plaque retention and may provoke local hypersensitivity reactions, whereas highly polished titanium and surgical-grade stainless steel are generally considered more biocompatible and less likely to irritate the surrounding tissues [6,7].

Habitual manipulation of oral jewelry is another major behavioral risk factor. Many individuals frequently move, bite, or play with their tongue or lip jewelry, often unconsciously. These repetitive habits substantially increase the mechanical forces applied to the gingival tissues and adjacent teeth, thereby accelerating gingival recession, connective tissue destruction, and localized bone loss [5]. Clinical observations indicate that patients who habitually manipulate their jewelry experience significantly more periodontal complications than those who leave the jewelry relatively undisturbed [5,6,8,23,27].

Poor oral hygiene is among the most significant modifiable contributors to periodontal damage in individuals with oral piercings. Oral jewelry creates additional plaque-retentive surfaces that facilitate bacterial adhesion and biofilm maturation while making routine plaque removal more difficult [6]. Persistent accumulation of bacterial biofilm around the jewelry and adjacent teeth leads to chronic gingival inflammation, increased bleeding on probing, and progressive periodontal destruction. When chronic inflammation is superimposed on repeated mechanical trauma, periodontal breakdown occurs more rapidly than with either factor alone. Therefore, individuals with inadequate plaque control exhibit a substantially higher risk of gingival recession, attachment loss, periodontal pocket formation, and alveolar bone resorption [9,24,34,35,36].

Several host-related anatomical and biological factors also influence susceptibility to periodontal injury. Individuals with a thin periodontal phenotype possess thinner gingival tissues and a thinner facial alveolar bone plate, making them more vulnerable to soft tissue recession and attachment loss following chronic mechanical irritation [33,47]. Likewise, patients with a narrow zone of keratinized gingiva, pre-existing gingival recession, orthodontically proclined mandibular incisors, or reduced alveolar bone thickness are at greater risk because their periodontal tissues provide less resistance to repeated trauma [7].

Smoking and other lifestyle factors might plausibly promote the periodontal effects of oral piercings. Tobacco use impairs gingival blood flow, suppresses immune function, delays wound healing, and increases the production of tissue-destructive inflammatory mediators, thereby accelerating periodontal attachment and bone loss [9,13]. The combination of smoking and chronic trauma from oral jewelry has been associated with more severe periodontal destruction than either risk factor alone. Additional behaviors, such as poor dietary habits, alcohol misuse, and irregular attendance for professional dental care, may further compromise periodontal health by reducing the body’s ability to control inflammation and maintain adequate oral hygiene. However, smoking cessation and appropriate diabetes management are measures supporting overall periodontal and systemic health rather than interventions proven to reduce piercing-specific periodontal complications [4].

Periodontal damage associated with oral piercings is strongly influenced by the interaction of these various risk factors rather than by the piercing alone. Prolonged wearing time, jewelry characteristics, plaque accumulation, and individual susceptibility all contribute to adverse periodontal outcomes [8,9]. These findings highlight the importance of comprehensive risk assessment and individualized patient counseling before and after oral piercing placement.

## 5. Other Oral Complications Associated with Oral Piercing

In addition to its detrimental effects on periodontal tissues, oral piercing has been associated with a wide spectrum of local and systemic complications affecting both the hard and soft tissues of the oral cavity. These complications range from minor transient problems occurring immediately after the piercing procedure to severe, irreversible conditions that may compromise oral function and general health [4,6]. The frequency and severity of these adverse effects are influenced by the location of the piercing, the type and material of the jewelry, the experience of the practitioner, oral hygiene practices, and individual host factors. It is indicated that tongue and lower lip piercings have been associated with the highest incidence of complications because of their continuous contact with the teeth, gingiva, and oral mucosa [8,9].

One of the most frequently reported non-periodontal complications is dental trauma, particularly enamel cracks, tooth fractures, cusp fractures, and excessive tooth wear. Continuous contact between metallic jewelry and the teeth during speaking, chewing, or unconscious habits such as playing with the jewelry generates repeated impact forces that progressively damage the enamel [5,10]. Initially, such trauma may present as superficial enamel fissures or abrasions; however, repeated impact can result in dentin exposure, pulp involvement, and complete crown fractures requiring restorative or endodontic treatment [6,8,19,48,49]. Mandibular incisors are particularly susceptible because they are in direct contact with tongue barbells and lower lip jewelry. Clinical investigations have demonstrated significantly higher rates of chipped teeth and enamel fractures among individuals with tongue piercings compared with non-pierced controls [19,20,48].

Soft tissue injuries are another common consequence of oral piercings. Immediately after placement, patients frequently experience pain, edema, bleeding, erythema, and localized inflammation as part of the normal healing process [1]. However, prolonged mechanical irritation from the jewelry may lead to chronic traumatic ulceration, frictional keratosis, mucosal erythema, hyperplastic tissue formation, fibrous scars, and localized granulomatous reactions [5]. Persistent trauma may also contribute to mucoceles and hypertrophic scar formation, particularly in lip piercings. In some individuals, prolonged irritation stimulates excessive fibrous tissue proliferation around the piercing tract, making oral hygiene difficult and increasing discomfort during mastication and speech [6,14,50].

Infectious complications represent another significant concern because oral piercings create a direct pathway for microorganisms to enter the underlying tissues and bloodstream. The oral cavity has a diverse microbiota, and the piercing wound provides an opportunity for bacterial colonization during the healing phase. Local infections may manifest as pain, swelling, erythema, purulent discharge, abscess formation, and delayed wound healing [1].

Neurological and functional complications may also occur following oral piercing. Trauma to sensory nerves during piercing can produce temporary or permanent paresthesia, dysesthesia, altered taste sensation, or numbness of the tongue and surrounding tissues [5]. Excessive post-operative tongue swelling may interfere with swallowing, speech, and airway patency, occasionally requiring emergency medical intervention. Patients frequently report transient difficulties with articulation, mastication, and swallowing during the healing period, while persistent speech alterations may develop when oversized jewelry interferes with normal tongue movement [1]. Chronic tongue edema may also increase the likelihood of accidental biting and further mucosal trauma [1,5].

Another clinically important complication is hypersensitivity and allergic reactions to the metallic components of oral jewelry. Nickel-containing alloys are among the most common causes of contact hypersensitivity and may induce localized erythema, edema, burning sensations, oral lichenoid reactions, and chronic mucosal inflammation [7]. Although titanium, niobium, and surgical-grade stainless steel exhibit superior biocompatibility, corrosion of metallic jewelry within the oral environment may release metal ions capable of provoking inflammatory responses in susceptible individuals [7].

The current evidence indicates that most of these complications could be preventable through appropriate patient selection, strict aseptic technique, careful jewelry selection, and regular professional follow-up. Many individuals undergo oral piercing without receiving adequate information regarding the potential oral and systemic risks. Consequently, dental professionals play a fundamental role in educating patients before and after piercing placement, emphasizing meticulous oral hygiene, the use of biocompatible jewelry, avoidance of repetitive jewelry manipulation, routine dental examinations, and early management of complications. Such preventive measures can substantially reduce morbidity and help preserve both oral and systemic health [5,8].

Other oral complications related to oral piercings are presented in Table 2.

## 6. Prevention of Oral Complications Associated with Oral Piercing

Prevention is the most effective strategy for minimizing the oral and periodontal complications associated with oral piercings, as many of these adverse effects are avoidable through appropriate patient education, careful jewelry selection, meticulous oral hygiene, and regular professional monitoring. Although oral piercings are widely used as a form of body modification, studies indicate that many individuals undergo piercing without receiving adequate information regarding possible short- and long-term oral complications, including gingival recession, clinical attachment loss, tooth fracture, infection, and localized periodontal destruction [5,6,8]. Preventive counseling should therefore ideally begin before piercing placement and continue throughout the period in which oral jewelry is worn, with dental professionals playing an important role in risk assessment, education, surveillance, and early management of complications [5,6,8].

An important component of prevention is ensuring that the piercing is performed by an appropriately trained practitioner using strict infection-control and aseptic procedures to reduce local/systemic infectious complications. Sterile single-use needles, properly sterilized instruments, appropriate hand hygiene, clean working surfaces, and adherence to established infection-control principles reduce the risk of local infection and transmission of blood-borne pathogens [6,8]. A thorough medical history should also be obtained before piercing to identify conditions that may increase the consequences of infection, bleeding, or impaired wound healing, including poorly controlled diabetes mellitus, immunosuppression, bleeding disorders, and selected cardiovascular conditions. Individuals with substantial medical risk may require medical consultation before undergoing an oral piercing procedure [5].

Jewelry characteristics are another important consideration because size, position, material, and duration of wear may influence the risk of oral injury. Long or poorly fitted barbells can increase the frequency of contact with teeth and periodontal tissues, while prolonged wear has been associated with greater prevalence of gingival recession and dental trauma [19,23,35,46]. After initial swelling has resolved and healing has progressed sufficiently, an excessively long starter barbell may be replaced by appropriately fitted jewelry under professional guidance to reduce unnecessary movement and repeated tissue contact. Jewelry should also have a smooth surface and be manufactured from materials suitable for prolonged intraoral exposure. Titanium and other appropriately manufactured biocompatible materials are generally preferred when metal hypersensitivity or corrosion is a concern, whereas nickel-containing alloys may provoke hypersensitivity reactions in susceptible individuals. Nevertheless, jewelry material alone does not eliminate the risks of mechanical trauma or microbial colonization but might reduce local tissue irritation and dental plaque accumulation, and consequently restrain periodontal complications [7,35].

Effective plaque control is particularly important because oral jewelry provides an additional non-shedding surface on which bacterial adhesion and biofilm formation can occur. Microbiological studies have demonstrated biofilm formation and colonization by potentially periopathogenic microorganisms on tongue-piercing jewelry and at piercing sites [34,35,36]. Patients should therefore maintain a thorough twice-daily tooth brushing with fluoride toothpaste, clean the jewelry and surrounding tissues without causing trauma, and use appropriate interdental cleaning methods. Antiseptic mouth rinses, including chlorhexidine-containing products, may be considered for short-term use during the early healing period when clinically indicated, but prolonged or routine use should be based on professional advice because chlorhexidine can cause adverse effects such as staining and altered taste. Proper plaque control may reduce local gingival inflammation leading to periodontal pocket formation and clinical attachment loss, although it cannot completely prevent trauma caused by repeated contact between jewelry and oral tissues [34,35,36].

Behavioral modification is also important in limiting mechanical complications. Patients should be advised to avoid repeatedly clicking, biting, chewing, or playing with tongue or lip jewelry because these behaviors increase repetitive contact between the jewelry and teeth or periodontal tissues and may contribute to enamel chipping, tooth fracture, gingival recession, and localized periodontal injury [5,6,19,41]. During healing, patients should also avoid behaviors known to impair tissue repair or increase irritation, particularly smoking. Tobacco use is an established modifier of periodontal inflammation, wound healing, and periodontal disease progression and may increase overall susceptibility to adverse periodontal outcomes in oral-piercing users, although a specific synergistic interaction between smoking and piercing trauma has not been conclusively demonstrated [6].

Regular professional dental examinations are important for early detection of piercing-associated complications. Clinical monitoring should include inspection of tooth surfaces and restorations; evaluation of plaque accumulation and gingival inflammation; bleeding on probing; probing pocket depth; gingival recession; clinical attachment levels; tooth mobility; and assessment of adjacent oral tissues. Radiographic examination may be indicated when clinical findings suggest attachment loss, osseous defects, or other hard-tissue pathology. Early detection of localized recession, increasing probing depths, attachment loss, or dental trauma permits intervention before more extensive irreversible damage develops. Professional plaque removal and repeated oral-hygiene instruction may also be beneficial when plaque accumulation is present [9,20,26,35].

Particular attention should be given to individuals with local or systemic factors that may increase their susceptibility to periodontal breakdown. A narrow zone of keratinized gingiva has been associated with greater buccal recession in individuals with lower-lip piercings, and unfavorable periodontal anatomy may reduce the tissues’ resistance to repeated mechanical irritation [12]. Thin periodontal phenotype reduced alveolar bone thickness, pre-existing recession, tooth malposition, and previous periodontal attachment loss may also increase vulnerability to recession in the presence of chronic traumatic forces, although these associations are better established in the general periodontal literature than specifically in piercing users [47,51]. Smoking and poorly controlled diabetes mellitus are established modifiers of periodontal disease and should therefore be incorporated into individualized risk assessment, even though their specific interaction with oral-piercing trauma remains incompletely defined [13].

Overall, prevention of oral-piercing complications is best approached through coordinated patient education, infection-control measures, appropriately designed jewelry, effective plaque control, avoidance of traumatic manipulation, and regular dental monitoring. Consequently, preventive recommendations should emphasize risk reduction rather than implying that all complications can be completely avoided. When progressive gingival recession, clinical attachment loss, persistent inflammation, severe dental trauma, or other significant complications develop, modification or removal of the jewelry together with appropriate dental or periodontal treatment should be considered to limit further tissue damage [5,6,8,9,19,20].

**Figure 2:**
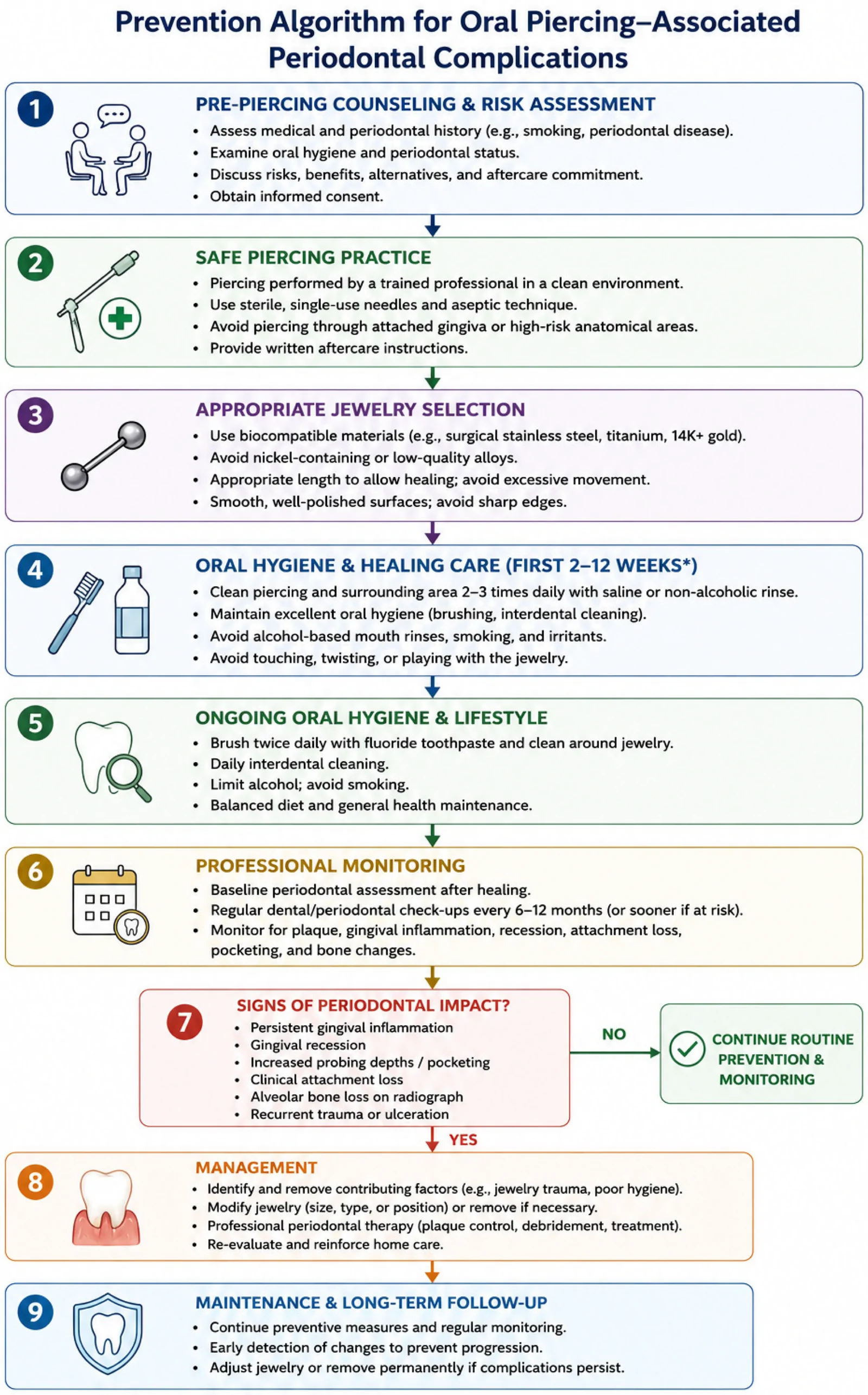
Preventive strategies for reducing oral and periodontal complications associated with oral piercings.

Preventive strategies for reducing oral and periodontal complications associated with oral piercings are presented on Figure 2.

## 7. Systemic Complications Associated with Oral Piercing

Although most adverse effects associated with oral piercings are confined to oral and perioral tissues, rare systemic complications have been reported and may be potentially life-threatening. Disruption of the highly colonized oral mucosa during piercing creates a portal through which microorganisms may enter deeper tissues and, potentially, the bloodstream, particularly during the initial healing period or in the presence of local infection [50]. Although transient bacteremia is generally managed efficiently in healthy individuals, patients with certain predisposing cardiac conditions may be at increased risk of infective endocarditis if circulating microorganisms adhere to susceptible endocardial surfaces. Several cases of infective endocarditis temporally associated with tongue or other body piercings have been reported [52,53].

Other serious infectious complications have also been described following oral or tongue piercing. Case reports include cerebral or cerebellar abscesses, deep neck-space infections, Ludwig’s angina, sepsis, and other disseminated infections arising after piercing-related microbial inoculation or local infection [50,54]. However, these complications should not be interpreted as common outcomes of oral piercing but rather as uncommon events requiring prompt medical recognition and treatment.

Inadequate sterilization of piercing equipment and poor infection-control practices may additionally provide a route for transmission of blood-borne pathogens. Hepatitis B virus (HBV), hepatitis C virus (HCV), and human immunodeficiency virus (HIV) are recognized as potential concerns in body-piercing procedures performed with contaminated or inadequately sterilized instruments [6]. However, direct evidence linking these infections specifically to oral piercing is limited; therefore, they are more appropriately described as potential infection-control risks rather than frequently documented oral-piercing complications.

Systemic allergic or hypersensitivity reactions represent another potential concern. Nickel-containing jewelry is a well-established cause of contact hypersensitivity, and susceptible individuals may develop localized or, less commonly, more extensive inflammatory reactions following exposure to nickel-containing alloys. Selection of appropriately manufactured biocompatible jewelry may therefore reduce, although not eliminate, material-related adverse reactions [7].

The likelihood and clinical consequences of infectious or systemic complications may be influenced by several host and procedural factors, including inadequate aseptic technique, poor aftercare, local infection, immunosuppression, poorly controlled diabetes mellitus, and underlying cardiovascular disease [7]. Accordingly, prevention should include careful medical history assessment, appropriate patient counseling, use of sterile single-use or properly sterilized equipment, adherence to rigorous infection-control procedures, selection of suitable jewelry, and prompt evaluation of persistent swelling, fever, purulent discharge, spreading infection, or other systemic symptoms. Individuals with significant cardiovascular or immunologic risk should obtain appropriate medical or dental advice before undergoing elective oral piercing [7,50].

## 8. Conclusions

Oral piercing has become an increasingly popular form of body modification, particularly among adolescents and young adults, despite its well-documented association with numerous oral and periodontal complications. Current evidence indicates that tongue and lower lip piercings pose risk to periodontal health because of their continuous mechanical interaction with the gingival tissues and adjacent teeth. Oral piercings have been associated with an increased prevalence of gingival recession, gingival inflammation, clinical attachment loss, periodontal pocket formation, and localized alveolar bone loss. These periodontal changes could be primarily attributed to the combined effects of chronic mechanical trauma, bacterial plaque accumulation, and sustained inflammatory responses, all of which contribute to the progressive destruction of the supporting periodontal tissues. Rare serious systemic infectious events, including infective endocarditis and deep or disseminated infections, have also been described; however, these observations are derived predominantly from case reports and do not permit estimation of incidence or causal risk.

Overall, the available literature demonstrates that oral piercing is not merely a cosmetic practice but a potential risk factor for significant oral and periodontal disease. The adverse effects associated with oral piercings could be minimized with proper preventive strategies. Patient education before piercing placement, adherence to strict aseptic techniques, selection of appropriately sized biocompatible jewelry, maintenance of excellent oral hygiene, avoidance of traumatic habits, and regular dental and periodontal examinations are fundamental measures for reducing the incidence and severity of complications. Dental professionals have a crucial role in educating patients about these risks, providing evidence-based preventive advice, and monitoring pierced individuals for early signs of periodontal deterioration. Through increased awareness, preventive care, and regular professional follow-up, many of the adverse oral consequences of oral piercing can be minimized, thereby improving both periodontal health and overall quality of life.

Although conducted studies consistently support an association between oral piercings and periodontal complications, the strength of these conclusions is limited by the characteristics of the available evidence. Much of the literature consists of small cross-sectional and case–control studies, supplemented by case series and case reports, with substantial heterogeneity in participant selection, piercing characteristics, exposure duration, periodontal assessment, and adjustment for potential confounders. These limitations restrict causal inference and make it difficult to determine the incidence, magnitude, and temporal progression of piercing-associated periodontal damage.

Future research should therefore prioritize prospective longitudinal cohort studies in which periodontal status is documented before or soon after piercing placement and reassessed at standardized intervals. Core periodontal outcomes should include gingival recession depth and width, clinical attachment level, probing pocket depth, bleeding on probing, plaque and gingival indices, and, where indicated, standardized radiographic measures of alveolar bone level. Studies should also consistently document piercing location, jewelry design, dimensions and material, duration of wear, habitual manipulation, oral hygiene practices, smoking status, periodontal phenotype, and baseline periodontal health. Such standardization would improve comparability between studies and facilitate more reliable quantitative synthesis.

Once major risk modifiers are better defined, intervention studies should determine whether targeted strategies—including modification of jewelry dimensions or material, structured oral-hygiene protocols, behavioral interventions to reduce jewelry manipulation, smoking cessation, and scheduled periodontal surveillance—can possibly prevent or limit tissue damage. Establishing standardized outcome measures and prospective study designs will be essential for moving the evidence base from predominantly associative observations toward clinically actionable estimates of risk and evidence-based preventive recommendations.

## Figures and Tables

**Table 1:** Summary of the impact of oral piercing on periodontal tissues.

Periodontal Effect	Short Description	Main Mechanism	Key References
Gingival recession	Most common periodontal complication; mainly affects the mandibular anterior teeth adjacent to tongue and lower lip piercings.	Chronic mechanical trauma from repeated contact between jewelry and gingiva.	Hennequin-Hoenderdos et al. (2016) [8]; Plastargias et al. (2014) [5]; Difloe-Geisert et al. (2024) [9]
Clinical attachment loss (CAL)	Irreversible loss of periodontal attachment that may compromise tooth support.	Persistent trauma combined with plaque-induced inflammation and connective tissue destruction.	Hennequin-Hoenderdos et al. (2016) [8] Pires et al. (2010) [20] Plessas et al. (2012) [23]
Periodontal pocket formation	Increased probing depths around teeth adjacent to oral piercings.	Apical migration of the junctional epithelium following inflammation and attachment loss.	Difloe-Geisert et al. (2024) [9] Schmidt et al. (2019) [26] Brooks et al. (2003) [27] Kapferer et al. (2008) [28]
Gingival inflammation	Characterized by redness, swelling, bleeding on probing, and increased plaque accumulation.	Bacterial biofilm retention around jewelry and continuous soft tissue irritation.	Plastargias et al. (2014) [5]; Campbell et al. (2002) [19] Pires et al. (2010) [20] Plessas et al. (2012) [23]
Alveolar bone loss	Localized resorption of supporting bone in severe or long-standing cases.	Chronic inflammation and osteoclastic bone resorption are secondary to trauma and periodontal infection.	Hennequin-Hoenderdos et al. (2016) [8]; Difloe-Geisert et al. (2024) [9] Levin et al. (2007) [41]

**Table 2:** Other oral complications associated with oral piercing.

Oral Complication	Short Description	Main Cause	Key References
Tooth fractures	Chipping or fracture of enamel, dentin, or cusps, particularly in mandibular incisors.	Repeated impact of metal jewelry against teeth.	Campbell et al. (2002) [19]; Plastargias et al. (2014) [5]
Enamel cracks and tooth wear	Development of enamel fissures, abrasions, and accelerated tooth wear.	Continuous friction between jewelry and tooth surfaces.	Campbell et al. (2002) [19]; Mosaddad et al. (2023) [6]
Dentin hypersensitivity	Sensitivity to thermal, tactile, or chemical stimuli following enamel loss or gingival recession.	Exposure of dentin due to trauma or recession.	Plastargias et al. (2014) [5] Difloe-Geisert et al. (2024) [9]
Mucosal ulceration	Painful ulcers and traumatic lesions affect the tongue, lips, or cheeks.	Constant irritation from oral jewelry.	López-Jornet et al. (2006) [1]; Plastargias et al. (2014) [5]
Edema and pain	Swelling and discomfort, particularly during the initial healing period.	Tissue trauma and inflammatory response after piercing.	López-Jornet et al. (2006) [1]; Malcangi et al. (2023) [4]
Bleeding	Localized bleeding immediately after piercing or following repeated trauma.	Injury to soft tissues and blood vessels.	López-Jornet et al. (2006) [1]; Mosaddad et al. (2023) [6]
Local infection	Pain, swelling, erythema, purulent discharge, and delayed healing.	Bacterial contamination during or after piercing.	Hennequin-Hoenderdos et al. (2016) [8]; Mosaddad et al. (2023) [6]
Speech and swallowing difficulties	Temporary or persistent impairment of speech, chewing, and swallowing.	Tongue swelling and interference from jewelry.	López-Jornet et al. (2006) [1]; Plastargias et al. (2014) [5]
Taste disturbances	Altered or diminished taste sensation.	Trauma to lingual nerves during piercing.	Plastargias et al. (2014) [5]
Nerve injury (Paresthesia)	Temporary or permanent numbness and altered sensation of the tongue or lips.	Damage to sensory nerves during piercing.	Plastargias et al. (2014) [5] López-Jornet et al. (2006) [1]
Allergic reactions	Contact hypersensitivity presents as erythema, burning, or oral lichenoid lesions.	Nickel or other metal components in jewelry.	Masood et al. (2023) [7]
Metal ion release	Chronic mucosal irritation and inflammatory reactions.	Corrosion of metallic jewelry in the oral environment.	Masood et al. (2023) [7]
Hyperplastic tissue formation	Fibrous tissue overgrowth around the piercing site.	Chronic irritation and prolonged inflammation.	Plastargias et al. (2014) [5]; Malcangi et al. (2023) [4]

## Data Availability

No new data were created or analyzed in this study. Data sharing is not applicable to this article.
